# Bacterial Community Changes in Early-Stage Engineering Simulation of Red Mud/Phosphogypsum-Based Artificial Soil Vegetation Restoration

**DOI:** 10.3390/biology14081020

**Published:** 2025-08-08

**Authors:** Yong Liu, Binbin Xue, Hefeng Wan, Lishuai Zhang, Zhi Yang, Jingfu Wang, Lirong Wang, Xiaohong Lin

**Affiliations:** 1 College of Biological and Environmental Engineering, Guiyang University, Guiyang 550005, China; 2 State Key Laboratory of Environmental Geochemistry, Institute of Geochemistry, Chinese Academy of Sciences (IGCAS), Guiyang 550081, China; 3 Guizhou Institute of Biology, Guiyang 550009, China

**Keywords:** artificial soil, bacterial community, phosphogypsum, red mud, soil development

## Abstract

Preparing red mud/phosphogypsum-based artificial soils for vegetation restoration is promising. However, how artificial soil develops during vegetation restoration is unclear, especially regarding the relationship between the bacterial community and the development of artificial soil. The bacterial community changes in the early-stage engineering simulation of red mud/phosphogypsum-based artificial soil vegetation restoration were analyzed for the first time in this paper. The results showed that the structure of the bacterial community was simple at the beginning, mainly consisting of Proteobacteria, Firmicutes, and Bacteroidota, with total abundances of 74.5% and 89.3% in the two plots, respectively. The richness, diversity, and evenness of the bacterial communities all significantly increased over time (*p* < 0.05), indicating that the compositions of the bacterial communities in artificial soils undergo constant development, adjustment, and optimization. There were good correlations between bacterial communities and environmental factors (e.g., pH, W_H2O_, OM, TN, TK, AK, TP), which generally reflected the significant synergistic development and interaction between the quality of the soil environmental and bacterial communities. There were complex dynamic changes in the functions of the bacteria during the development of artificial soils, which were mainly reflected in the decline in the abundances of chemoheterotrophy, aerobic chemoheterotrophy, and animal parasites or symbionts, but there was an increase in the abundances of phototrophy, cyanobacteria, and dark sulfide oxidation. This reflects the highly active physiological and biochemical reaction functions of bacterial communities in the development of artificial soils, which is of great significance for continuously enhancing the fertility quality and ecological attributes of artificial soils.

## Simple Summary

Preparing red mud/phosphogypsum-based artificial soil for vegetation restoration is promising. This study is the first to report on the changes in the composition and function of bacterial communities in early-stage vegetation restoration and their relationship with the quality of the soil environment. We indicate that the bacterial communities changed from simple initial species to more diverse ones, and they were constantly changing in order to achieve optimization. The co-development between the bacterial communities and the quality of the artificial soil environment was very clear. The bacterial communities exhibited complex physiological and biochemical functional changes that play an important role in enhancing the comprehensive fertility and ecological attributes of artificial soil.

## 1. Introduction

Red mud and phosphogypsum are bulk industrial byproducts produced in large quantities by the electrolytic aluminum and phosphorus chemical industries, respectively. They have been stockpiled in numerous yards for a long period of time because there is a lack of effective disposal methods. Historically, the global stocks of red mud and phosphogypsum reached 4 and 7 billion tons, respectively, with annual increments of more than 120 and 250 million tons [1,2]. Red mud and phosphogypsum both contain many harmful elements, posing great risks of polluting the surrounding environment and to biological health [3,4]. In the case of poor management, red mud and phosphogypsum yards can collapse due to rainstorms and geological disasters, seriously threatening the safety of nearby people and environments [5,6]. The disposal of red mud and phosphogypsum has attracted lots of international attention, and there is an urgent need for large-scale, low-cost, and efficient utilization of the disposal of these byproducts [7,8]. At present, red mud and phosphogypsum are utilized in limited quantities, with comprehensive utilization rates of only about 15% and 40%, respectively [1,9]. Recently, the preparation of red mud/phosphogypsum-based artificial soils for vegetation restoration in mining areas and other soil-deficient areas has become a highly promising solution that could effectively utilize large amounts of red mud and phosphogypsum [10,11]. Red mud and phosphogypsum are the main components of artificial soil, along with a small amount of auxiliary materials, which results in the formation of soil-like substrates that are suitable for plant adaptability and friendly growth; there is no need to precisely control the technical conditions, as is the case with chemical production. By allowing for the large-scale and low-cost synergistic consumption of red mud and phosphogypsum, this would turn bare yards into natural ecological landscapes.

Microorganisms are widely present in soil and have always been a key factor in the environmental quality of soil; they play a vital role in the decomposition of soil organic matter, the conversion and cycling of soil nutrients, and the improvement in soil structure [12]. There is a huge quantity of bacteria, as one of the main taxa of microorganisms, and they have a strong adaptability. They can colonize in barren soil relatively early, and they play an essential role in driving soil development [13]. Since it is merely a simple mixture of different materials, the physical structure, physicochemical properties, and different nutrients of newly prepared red mud/phosphogypsum-based artificial soil are heterogeneous, and thus it has almost no ecological attributes. Bacteria can adapt and serve as pioneer organisms to colonize environments and promote the conversion and stable equilibrium of nutrients in artificial soil, contributing to its continuous development and maturation. On the contrary, driven by the development of artificial soil, the continuous changes in the environmental quality of soil also regulate the composition of bacterial communities, which leads to alterations in microbial diversity, ecological functions, and the soil ecosystem [14]. However, since artificial soil solution is a newly proposed concept, the basic data and information are relatively lacking, and our theoretical understanding is insufficient. Additionally, the synergistic development relationship between artificial soils and bacterial communities remains unclear.

In this study, by using the same engineering simulation test site for red mud/phosphogypsum-based artificial soil vegetation restoration that we previously studied [11], a comprehensive analysis of the changes in bacterial community composition, the correlation between bacterial communities and soil environmental quality, and the changes in the functional abundance of bacterial communities at different times during early-stage artificial soil vegetation restoration was conducted. Our research objectives were to further provide data supplementation for the theoretical development of red mud/phosphogypsum-based artificial soil technology and to offer scientific evidence to develop a deeper understanding of bacterial communities’ composition and their functional changes in early-stage artificial soil vegetation restoration, as well as their impacts on the environmental quality of artificial soil.

## 2. Materials and Methods

### 2.1. Engineering Simulation of Artificial Soil Vegetation Restoration

Two different artificial soils (DK and JZ) were collated in an open space of about 30 m^2^. The main materials for preparing the artificial soils, red mud and phosphogypsum, were collected from a red mud yard and a phosphogypsum yard in Guizhou, China, respectively, with pH values of 11.7 and 2.4 and moisture contents of 15.2% and 16.3%. Other auxiliary materials included rice hull, distillers grain, bentonite, and polyacrylamide (Table 1). A small amount of yeast powder and a microbial agent mainly based on *Bacillus subtilis* were added to balance out the soils for 10 d, followed by the addition of 100 larvae of *Eisenia foetida*. A total of 38 different types of plant seedlings and seeds (e.g., wood, herbs, flowers, and succulents) were directly transplanted or scattered into DK and JZ plots and watered regularly to maintain a soil moisture content of about 30% in order to achieve natural growth and germination [11].

### 2.2. Sample Collection and Determination

Samples of artificial soils near the surface layer (8–15 cm) in the DK and JZ plots were collected at 30 and 150 d of the simulation of vegetation restoration, respectively, using the diagonal distribution method. Specifically, three points were set on the diagonal line of each plot as three parallel groups; samples were collected 3 times within a diameter of 30 cm at each point and mixed to make the final sample to be assessed for its common physicochemical parameters and to undergo major nutrients analysis. Two additional points (a total of five points) were added on the diagonal line of each plot, resulting in five parallel groups that were used to analyze the bacterial community. After freeze-drying, the removal of foreign bodies, and crushing, the quartering method was used to reduce the number of samples. The physicochemical parameters (moisture (W_H2O_), pH, organic matter (OM), and cation exchange capacity (CEC)) and major nutrients (total nitrogen (TN), total phosphorus (TP), total potassium (TK), alkaline nitrogen (AN), available phosphorus (AP), and available potassium (AK)) were analyzed. Details of the testing method we used are outlined in reference [11].

The composition of bacterial communities was determined using 16S rRNA gene high-throughput sequencing supplied by Shanghai Majorbio Bio-pharm Technology Co., Ltd. (Shanghai, China) (https://www.majorbio.com; accessed on 20 March 2025). The DNA concentration was determined using a NanoDrop-2000 (Thermo Fisher Scientific, Waltham, MA, USA), and the DNA integrity was detected via agarose gel electrophoresis (the samples were melted on ice, fully mixed, and centrifuged, and then an appropriate amount was run on a 1% agarose gel at 5 V/cm for 20 min). PCR amplification experiments were performed for the V3-V4 variable region of the 16S rRNA gene (PCR primer design: 338F-ACTCCTACGGGGAGGCAGCAG; 806R-GGACTACHVGGGTWTCTAAT). The formal PCR test used TransGen AP221-02: TransStart Fastpfu DNA Polymerase, with a 20 μL reaction system: 4 μL of 5×FastPfu Buffer, 2 μL of 2.5 mM dNTPs, 0.8 μL of Forward Primer (5 μM), 0.8 μL of Reverse Primer (5 μM), 0.4 μL of FastPfu Polymerase, 0.2 μL of BSA, 10 ng of Template DNA, adding ddH2O to a total of 20 μL. The initial denaturation was at 95 °C for 3 min, followed by 27 cycles of denaturing at 95 °C for 30 s, annealing at 55 °C for 30 s, and extension at 72 °C for 45 s, and there was a single extension at 72 °C for 10 min. The amplified products were used for library construction using the NEXTFLEX Rapid DNA-Seq Kit (NEXTFLEX, San Jose, CA, USA) and sequenced on the Illumina NextSeq 2000 high-throughput sequencing platform (Illumina, San Diego, CA, USA). Following acquisition of raw sequencing data, quality control and bioinformatics processing were performed sequentially: First, raw sequencing reads were filtered for quality using fastp (v 0.20.0) to remove low-quality sequences, adapter sequences, and other contaminants. Second, quality-controlled paired-end reads were assembled using FLASH (v 1.2.7) to obtain complete target sequences. Finally, operational taxonomic units (OTUs) were clustered using UPARSE (v 11.0.667) based on 97% sequence similarity, generating representative sequences for subsequent taxonomic classification and microbial community diversity analysis. The statistics of sequencing data of bacterial communities in artificial soil plots were detailed in Table S1 of the Supplementary Data.

### 2.3. Data Analysis

The bacterial communities and functions in artificial soil plots, as well as their correlations with different environmental factors, including Venn, bar plot, α-diversity, β-diversity (Principal coordinate analysis, PCoA), Mantel test, correlation heatmap, and function heatmap, were all analyzed and mapped on the online Majorbio Cloud Platform (www.majorbio.com, accessed on 20 March 2025) [15]. The Venn diagram constructed using jvenn (http://jvenn.toulouse.inra.fr/app/index.html, accessed on 20 March 2025) with shared and unique phyla, genera or OTUs was used to depict the similarity and difference among the bacterial communities. The PCoA based on Bray–Curtis distance and analysis of similarities (ANOSIM) to determine the similarity among the bacterial communities, and the Mantel tests based on Bray–Curtis distance matrix to correlate bacterial communities with environmental factors, were both analyzed by using vegan package in R (v3.3.1). Pearson correlation heatmaps were performed to estimate the relationships between bacterial phyla/genera and environmental factors using pheatmap package in R (v 3.3.1). The functional abundances in the bacterial communities were predicted using the functional annotation of prokaryotic taxa database (FAPROTAX_1.2.1 database).

## 3. Results

### 3.1. Changes in Composition of Bacterial Communities in the Artificial Soil Plots

**Figure 1:**
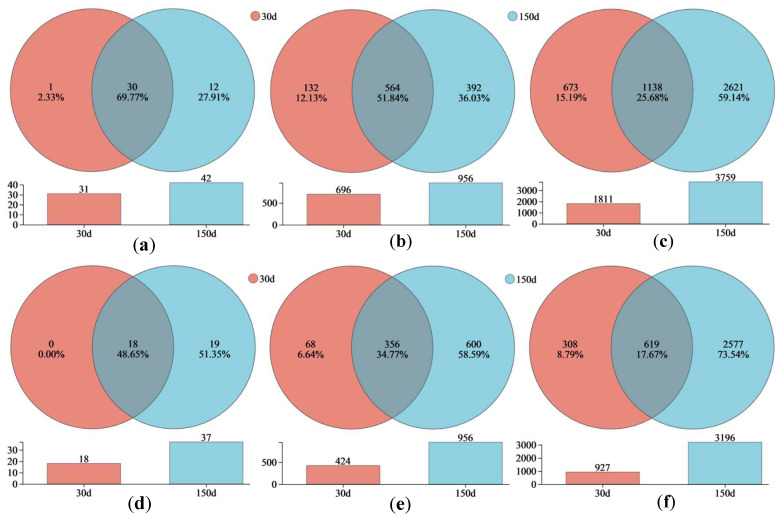
Venn analysis of bacterial communities in the artificial soil plots. Note: phyla/DK (**a**), genera/DK (**b**), OTUs/DK (**c**), phyla/JZ (**d**), genera/JZ (**e**), and OTUs/JZ (**f**).

As shown in Figure 1a–f, the number of bacterial phyla, genera, and operational taxonomic units (OTUs) in the two plots was relatively small, with the DK plot being significantly higher than the JZ plot in each case at 30 d. Over time, to 150 d, the number of bacterial phyla, genera, and OTUs in the two plots increased significantly, and the differences between the two plots narrowed significantly (overall, the DK plot was still slightly higher than the JZ plot). The number of bacterial OTUs in the DK and JZ plots increased from 1811 to 3759 (Figure 1c) and from 927 to 3196 (Figure 1f), respectively. The number of bacterial phyla in the DK and JZ plots increased from 31 to 42 (Figure 1a) and from 18 to 37 (Figure 1d), respectively. The number of bacterial genera in the DK and JZ plots increased from 696 to 956 (Figure 1b) and from 424 to 956 (Figure 1e), respectively. The new bacterial phyla and genera accounted for 51.4% and 58.6%, respectively, of the JZ plot, which were significantly higher than those of the DK plot.

**Figure 2:**
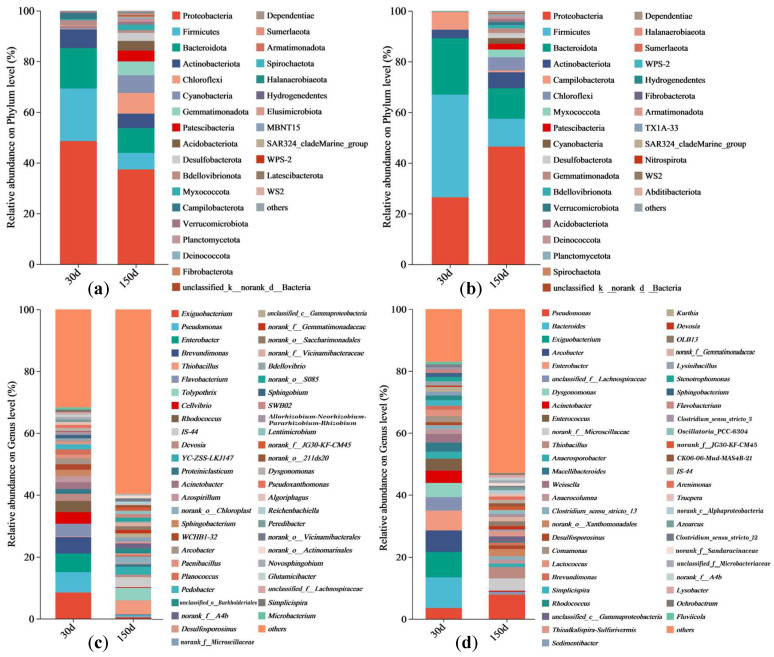
Composition of bacterial communities in the artificial soil plots. Note: phyla/DK (**a**), phyla/JZ (**b**), genera/DK (**c**), and genera/JZ (**d**); those with an abundance ranking lower than 30 (phyla) or 50 (genera) are combined into “others”.

In the DK plot, the bacterial phyla with an abundance > 5% at 30 d included Proteobacteria (48.5%), Firmicutes (20.7%), Bacteroidota (16.0%), and Actinobacteriota (7.4%), though their abundances all decreased to 37.4%, 6.5%, 9.8%, and 5.7%, respectively, at 150 d; at this time point, the new phyla with an abundance > 5% included Chloroflexi (8.1%), Cyanobacteria (7.1%), and Gemmatimonadota (5.4%) (Figure 2a). The bacterial genera with an abundance > 5% included *Exiguobacterium* (8.5%), *Pseudomonas* (6.5%), *Enterobacter* (6.0%), and *Brevundimonas* (5.4%) at 30 d, though their abundances all dropped to below 5% at 150 d. The three most abundant bacterial genera at 150 d were *Thiobacillus* (4.6%), *Tolypothrix* (4.0%), and *IS-44* (3.2%) (Figure 2c).

In the JZ plot, the bacterial phyla with an abundance > 5% at 30 d included Proteobacteria (26.5%), Firmicutes (40.5%), Bacteroidota (22.3%), and Campilobacterota (6.9%). The abundance of Proteobacteria (46.4%) increased significantly, while the abundances of Firmicutes (11.0%), Bacteroidota (12.1%), and Campilobacterota (0.7%) all decreased significantly. The new bacterial phyla with an abundance > 5% at this time point included Actinobacteriota (6.3%) and Chloroflexi (5.3%) (Figure 2b). At 30 d, the bacterial genera with an abundance > 5% included *Bacteroides* (9.9%), *Exiguobacterium* (8.2%), *Arcobacter* (6.8%), and *Enterobacter* (6.4%), though their abundances all dropped to below 5% at 150 d. At 150 d, only *Pseudomonas* (7.8%) had an abundance > 5% (Figure 2d).

### 3.2. Changes in Bacterial α-Diversity in the Artificial Soil Plots

As shown in Table 2, the Sobs, Ace, and Chao indices of the bacterial communities in the DK and JZ plots were all relatively small at 30 d, with significant differences between the two plots (DK > JZ; *p* < 0.05). At 150 d, these indices all increased significantly, with no significant difference between the two plots (*p* > 0.05). This indicated that the richness of the bacterial communities in the DK plot was significantly higher than that in the JZ plot at 30 d; this increased significantly in both plots at 150 d, when it then became similar. Compared with the JZ plot, the DK plot had a larger Shannon index and a smaller Simpson’s index at 30 d (*p* < 0.05). At 150 d, the DK and JZ plots each had a significant increase in the Shannon index and a significant decrease in Simpson’s index (*p* < 0.05), indicating that the diversity of the bacterial communities increased significantly in each plot, though that in the DK plot was still slightly higher. The Pielou_e index varied significantly between different plots and across different days (*p* < 0.05); it was lower at 30 d in the JZ plot compared with the DK plot, indicating lower evenness of the bacterial communities. At 150 d, the DK and JZ plots each had a significant increase in the Pielou_e index (*p* < 0.05), indicating that community evenness increased. The coverage index was >99.5% in each plot, indicating that almost all bacterial communities in the artificial soils were detected.

### 3.3. Changes in Bacterial β-Diversity in the Artificial Soil Plots

**Figure 3:**
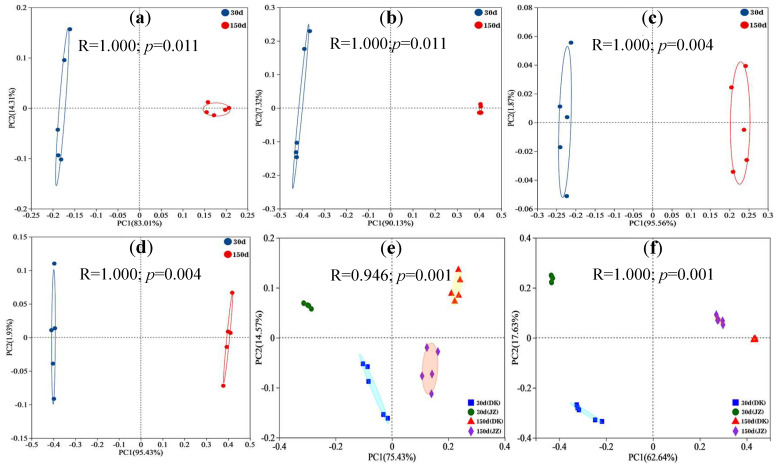
PCoA analysis of bacterial β-diversity in the artificial soil plots. Note: phyla/DK (**a**), genera/DK (**b**), phyla/JZ (**c**), genera/JZ (**d**), phyla/DK-JZ (**e**), and genera/DK-JZ (**f**).

The total variance in the bacterial phyla in the two plots (PC1 + PC2), measured using principal coordinate analysis (PCoA), reached as high as 97.32% and 97.43%, respectively (Figure 3a,c). At the genus level, the total variance explained (PC1 + PC2) using PCoA reached as high as 97.45% and 97.36%, respectively (Figure 3b,d). Meanwhile, Figure 3a–d outline the significant difference in the bacterial phyla and genera in the DK and JZ plots at 30 and 150 d, respectively; the parallel samples of bacterial phyla (or genera) in the two plots were scattered at 30 d but concentrated at 150 d (especially in the DK plot), reflecting the trend of the bacterial community being structured toward stabilization. The bacterial phyla or genera in the DK and JK plots were located in different zones at different times, indicating that there was a significant difference in the bacterial communities between the two plots (Figure 3e,f). The bacterial genera zones in the two plots were closer at 150 d than at 30 d, suggesting that the community compositions of bacterial genera in the two plots became more similar over time.

### 3.4. Correlation Between Bacterial Communities and Environmental Factors in the Artificial Soil Plots

**Figure 4:**
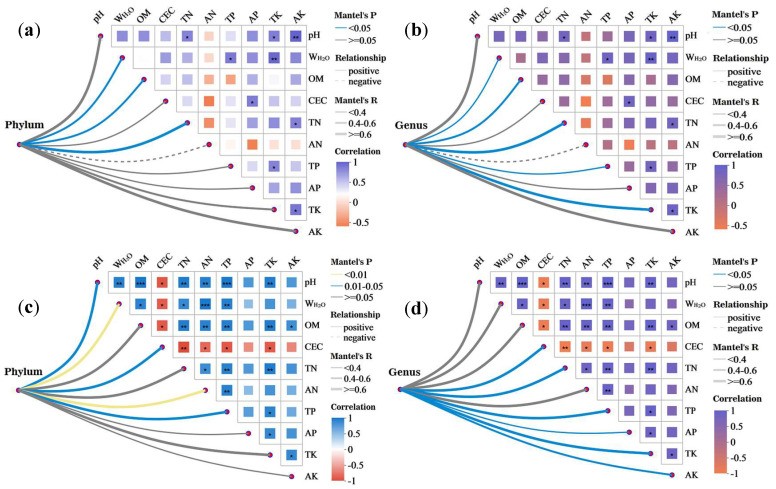
Mantel test correlation heatmap of bacterial communities in the artificial soil plots. Note: phyla/DK (**a**), genera/DK (**b**), phyla/JZ (**c**), and genera/JZ (**d**); Bray–Curtis, Pearson correlation; * 0.01 < *p* ≤ 0.05, ** 0.001 < *p* ≤ 0.01, *** *p* ≤ 0.001.

According to the Mantel test correlation analysis, the bacterial phyla and genera in the DK plot were positively correlated with all of the environmental factors (Table 3), except for AN (with which the bacterial phyla and genera were negatively correlated) (Figure 4a,b). The bacterial phyla were significantly positively correlated with W_H2O_, OM, and TN (*p* < 0.05), and the bacterial genera were significantly positively correlated with W_H2O_, OM, TN, TP, and TK (*p* < 0.05). In the JZ plot, the bacterial phyla and genera were positively correlated with all of the environmental factors, with the phyla showing highly significant positive correlations with W_H2O_ and AN (*p* < 0.01) and significant positive correlations with pH, CEC, and TP (*p* < 0.05). Meanwhile, the genera were significantly positively correlated with CEC, TN, TP, AP, TK, and AK (*p* < 0.05) (Figure 4c,d). There were low correlations between all of the environmental factors in the DK plot, except for a few that had significant or highly significant positive correlations (Figure 4a,b). In the JZ plot, there were highly significant positive correlations between all of the environmental factors (*p* < 0.05–0.001), except for CEC, which had highly significant negative correlations with pH, W_H2O_, OM, TN, AN, TP, and TK (Figure 4c,d).

**Figure 5:**
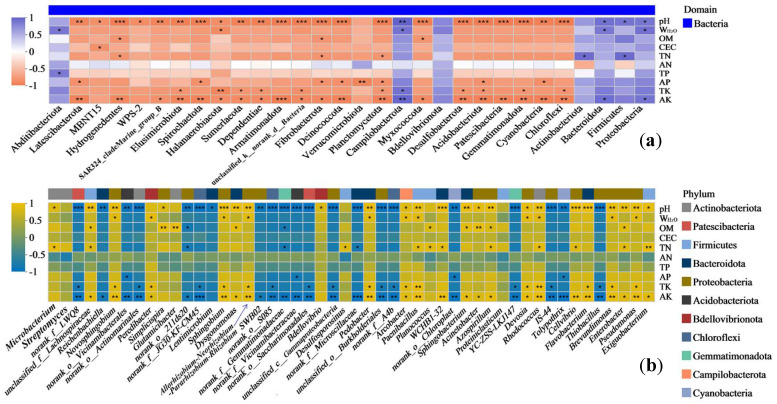
Correlation heatmap of bacterial communities in DK plot. Note: phyla (**a**) and genera (**b**); * 0.01 < *p* ≤ 0.05, ** 0.001 < *p* ≤ 0.01, *** *p* ≤ 0.001.

In the DK plot, the bacterial phyla with relatively high abundances (Proteobacteria, Firmicutes, Bacteroidota, and Actinobacteriota) were positively correlated with the majority of environmental factors (especially pH, W_H2O_, TN, and AK) (Figure 5a). Proteobacteria and Bacteroidota were significantly positively correlated with pH, W_H2O_, and AK (*p* < 0.05), and Firmicutes and Actinobacteriota were significantly positively correlated with TN (*p* < 0.05). Most phyla with relatively low abundances were mainly negatively correlated with different environmental factors, showing highly significant negative correlations with pH, TK, and AK (*p* < 0.05–0.001; Figure 5a). The bacterial genera with relatively high abundances (*Exiguobacterium*, *Pseudomonas*, *Enterobacter*, and *Brevundimonas*) were positively correlated with the majority of environmental factors (such as pH, AK, W_H2O_, OM, TN, and TK), showing significant or highly significant positive correlations with pH and AK (*p* < 0.05–0.01; Figure 5b). Most genera with relatively low abundances had highly significant positive or negative correlations with pH, AK, OM, TK, W_H2O_, TN, and AP (*p* < 0.05–0.001; Figure 5b).

**Figure 6:**
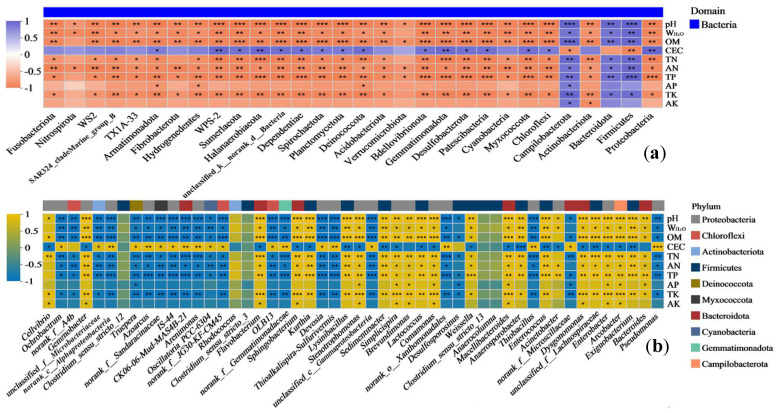
Correlation heatmap of bacterial communities in JZ plot. Note: phyla (**a**) and genera (**b**); * 0.01 < *p* ≤ 0.05, ** 0.001 < *p* ≤ 0.01, *** *p* ≤ 0.001.

In the JZ plot, most of the bacterial phyla had significant correlations with all of the environmental factors (except for AP and AK) (Figure 6a). The phyla with relatively high abundances (Firmicutes, Bacteroidota, and Campilobacterota) had highly significant positive correlations with the majority of environmental factors, while Proteobacteria and Actinobacteriota showed highly significant negative correlations with the majority of environmental factors. Most of the bacterial phyla with relatively low abundances were negatively correlated to varying degrees with all of the environmental factors, except for CEC (Figure 6a). Most of the bacterial genera in the JZ plot had highly significant correlations with all of the environmental factors (except for AP and AK) (Figure 6b). The high-abundance *Pseudomonas* was highly significantly negatively correlated with pH, W_H2O_, OM, TN, AN, TP, and TK (*p* < 0.05–0.01), but it was highly significantly positively correlated with CEC (*p* < 0.001). In contrast, *Bacteroides*, *Exiguobacterium*, *Arcobacter*, and *Enterobacter* showed highly significant positive correlations with pH, W_H2O_, OM, TN, AN, TP, and TK (*p* < 0.05–0.001), but it showed a highly significant negative correlation with CEC (*p* < 0.05–0.01; Figure 6b).

### 3.5. Changes in Bacterial Functional Abundance in the Artificial Soil Plots

**Figure 7:**
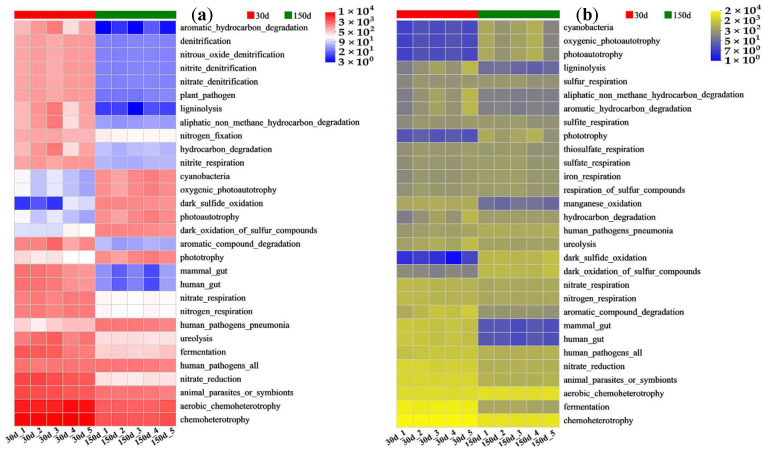
Function heatmap of bacterial communities in the artificial soil plots. Note: DK (**a**) and JZ (**b**).

The functional abundance of bacterial communities in the artificial soils of the two plots was predicted using FAPROTAX. The bacterial functions with relatively high abundances in the DK plot at 30 and 150 d all included chemoheterotrophy, aerobic chemoheterotrophy, and animal parasites or symbionts; these all declined somewhat at 150 d (Figure 7a). The abundances of most bacterial functions, such as nitrate reduction, mammal gut, human gut, nitrate respiration, and nitrogen respiration, also declined to varying degrees at 150 d. However, there was a significant increase in the abundances of bacterial functions such as human pathogens_pneumonia, dark oxidation of sulfur compounds, photoautotrophy, dark sulfide oxidation, oxygenic photoautotrophy, and cyanobacteria at 150 d. The JZ plot also showed substantial changes in the abundances of bacterial functions between 30 and 150 d (Figure 7b). The abundances of most of the bacterial functions, such as chemoheterotrophy, fermentation, animal parasites or symbionts, nitrate reduction, human pathogens_all, mammal gut, and human gut, declined to varying degrees at 150 d. However, there was a significant rise in the abundances of bacterial functions at 150 d, including the dark oxidation of sulfur compounds, dark sulfide oxidation, phototrophy, cyanobacteria, oxygenic photoautotrophy, and photoautotrophy.

## 4. Discussion

### 4.1. Development of Red Mud/Phosphogypsum-Based Artificial Soils in Vegetation Restoration

Red mud/phosphogypsum-based artificial soil has the notable feature of its nutrient distribution being extremely inhomogeneous, it has almost no ecological attributes, and it is still at the initial stage of soil development, although it is similar to natural soil in terms of its physicochemical properties and nutrient characteristics. Vegetation restoration in artificial soil is a process of continuous development, especially with the homogenization of soil nutrients and the significant enhancement of its ecological attributes [14,16]. In addition to artificial plants that produce various biological enzymes and root exudates to continuously enrich the soil material and improve soil physicochemical properties, some pioneering bacterial species with strong adaptability also rapidly colonize and grow sooner to promote the formation of homogeneous matrices through the fermentation, degradation, and fusion of materials in artificial soils; this improves the physicochemical conditions of artificial soils and facilitates the conversion of nutrients such as carbon, nitrogen, and phosphorus [11,17,18]. With the environmental quality of artificial soils improving, the structure of bacterial communities will be continuously optimized through constant adaptations, which will further enhance the diversity of bacterial communities and stabilize their structure [19]. In combination with the increasing inhabitation and diversification of other microorganisms (such as fungi) and arthropods, an artificial soil micro-ecosystem will take shape [20,21]. This is a key sign of the development and maturation of artificial soils and an important basis for evaluating their ecological reconstruction [20,22]. Meanwhile, as different artificial soils are prepared from different types of materials at different ratios, their physicochemical properties and nutrient characteristics often differ, meaning that vegetation restoration and ecological reconstruction processes are likely to differ as well [23,24,25]. Differences in the types of adaptable plants and microbial species lead to differences in the direction and speed of artificial soil development, as well as the formation of soil micro-environments and micro-ecosystems [26,27]. This is helpful for broadening the application scenarios and landscape types related to the vegetation restoration and ecological reconstruction of artificial soils.

### 4.2. Changes in Bacterial Communities in Early-Stage Artificial Soil Vegetation Restoration

The colonization, amplification, and diversification of bacterial communities constitute some of the most important changes in early-stage artificial soil vegetation restoration [28]. In this study, the material types and ratios of artificial soils in the DK and JZ plots were different, and their development was in the start-up stage at 30 d, with poor overall soil quality, which resulted in relatively low bacterial community richness, diversity, and evenness in both plots at this time, with significant differences in the composition of the bacterial communities. The dominant bacterial phyla in the two plots mainly included Proteobacteria, Firmicutes, and Bacteroidota, with combined abundances of 74.5% and 89.3% in the DK and JZ plots, respectively. These three dominant phyla generally have strong stress resistance, high saline–alkaline tolerance, and they are commonly found in decomposed and fermented environments, which coincided with the saline–alkaline characteristics of the artificial soils in the two plots and the fact that the added organic matter (such as rice hull and distillers grains) remained under degradation and decomposition [29,30,31,32,33,34,35]. The number of bacterial phyla, genera, and OTUs all increased significantly, which was particularly manifested in the increased overall richness, diversity, and evenness of the bacterial communities, which indicated that the environmental conditions of the two plots became better, allowing for more bacteria to grow and multiply well, meaning that the bacterial communities experienced constant adjustment and optimization. Due to the significant increase in the types and quantities of bacteria, the dominant positions of some previously abundant bacterial phyla or genera have declined, contributing to an improved balance among different bacterial species in artificial soils. The soil micro-ecosystem became more stable and the metabolic cycles and energy flow were more diverse, important signs of the continuous development and maturation of artificial soils [36,37]. There are obvious differences in the bacterial communities (especially the proportions of dominant bacteria) between the two plots, and these also differed greatly from our previous results of potted plants in artificial soils [38]. This indicates that there are great uncertainties in bacterial communities in artificial soils due to the fact that they are influenced by a combination of factors, such as the material composition of soil, development time, and the types of plants grown in different artificial soils [23,24,25].

### 4.3. Changing Relationships Between Bacterial Communities and Environmental Factors in Early-Stage Artificial Soil Vegetation Restoration

Generally, there is a mutual interaction that reinforces the relationship between the composition of bacterial communities and soil environmental quality [19,39]. Different environmental factors in soils, such as pH, moisture, organic matter, and nutrients, all affect the growth and metabolism of different bacteria, thereby influencing the composition and abundance of the bacterial communities [40]. Conversely, as one of the main functional biological groups in soil, bacteria play a crucial role in promoting the transformation of various nutrients in the soil through the decomposition, metabolism, and circulation of organic matter, thereby contributing to the improvement in soil environmental quality [41]. In this study, the bacterial phyla and genera in both plots were positively correlated with all of the environmental factors, except for AN (which was negatively correlated with the bacterial phyla and genera in the DK plot). This showed the mutual influence and obvious synergistic changes between the bacterial communities and environmental factors in the artificial soil plots. To a certain degree, bacterial communities are key contributors to the improvement in the quality of the artificial soil environment. The pH of artificial soil in the DK plot was significantly higher than that in the JZ plot; Proteobacteria, which prefer alkaline environments, had the highest abundance (up to 48.5%), and this was significantly higher than that in the JZ plot [32,42]. The fermentation and decomposition characteristics of artificial soil in the JZ plot were highlighted due to the addition of distillers grains; Firmicutes, which prefer such an environment, had the highest abundance (40.5%), and this was significantly higher than that in the DK plot [29,30]. These findings indicated that some characteristic bacterial species in the bacterial community serve as indicators of artificial soil environment conditions. The main dominant bacterial phyla (such as Proteobacteria, Firmicutes, Bacteroidota, and Actinobacteriota) and genera (such as *Exiguobacterium*, *Pseudomonas*, *Enterobacter*, and *Brevundimonas*) in the DK plot were positively correlated with almost all of the environmental factors, while most low-abundance bacterial phyla were negatively correlated with different environmental factors. This implied that due to the relatively unfavorable physicochemical conditions and nutrient-scarce environments of the primary artificial soils, very few bacterial phyla and genera with strong adaptability and nutritional competitiveness occupied dominant positions, played active roles in improving the physicochemical conditions of artificial soils, and promoted the increase in nitrogen, phosphorus, and potassium. Compared with the DK plot, the JZ plot showed more significant correlations between almost all of the bacterial phyla and genera with different environmental factors. Some dominant phyla and genera showed significant positive correlations with the majority of environmental factors, while others showed significant negative correlations. Similarly, most low-abundance bacterial phyla showed significant negative correlations with all of the environmental factors (except for CEC, AP, and AK). These results initially demonstrated the relationships of competition, trade-off, and dynamic balance between different bacterial phyla and genera under the influence of environmental factors in artificial soils. In particular, some dominant bacterial phyla (such as Firmicutes, Bacteroidota, and Campilobacterota) or genera (such as *Pseudomonas*) played positive roles in improving the physicochemical properties and nutrient content of artificial soils.

### 4.4. Changes in Bacterial Function in Early-Stage Artificial Soil Vegetation Restoration

Bacteria in the soil perform a wide range of functions, mainly including the ability to secrete different metabolites (such as organic acids) and produce various enzymes (such as hydrolases) to promote a series of processes such as the mineralization and humification of organic matter, nitrogen fixation, nitrification and denitrification, and phosphorus cycling and to ultimately enhance soil fertility [43,44,45,46]. When soil fertility gradually improves, the growth and metabolism of more bacterial communities becomes suitable, generating more bacterial functions. This creates a positive feedback effect, which further enhances overall soil fertility and enriches the types of bacterial communities and their functions [13,41]. The FAPROTAX prediction of bacterial functions with high abundances in the two plots in the early-stage artificial soil vegetation restoration all included chemoheterotrophy, aerobic chemoheterotrophy, and animal parasites or symbionts. Chemoheterotrophy and aerobic chemoheterotrophy might be related to the addition of rice husks and distillers grains in the two plots and their aerobic utilization as organic carbon sources via bacteria [47]. The presence of animal parasites or symbionts indicated that the two plots contained a high abundance of animal parasitic or pathogenic bacteria, which are obviously detrimental to the quality of the artificial soil environment. The three bacterial functions with the highest abundances all declined somewhat, and the relatively low-abundance mammal gut and human gut functions also declined significantly at 150 d. This indicated that the gradual degradation and reduction in organic substances such as rice husks and distillers grains in the artificial soils lead to the functions of the bacteria that utilize these substances as carbon sources to be weakened. It also showed that as the organic matter gradually decomposed and the abundance of beneficial bacteria increased, to a certain extent, this inhibited the pathogenic bacteria, and accordingly, their functions significantly decreased, which are important manifestations of a healthier soil environment and a better ecological balance [48,49]. Moreover, nitrogen cycling-related bacterial functions with relatively high abundances, such as nitrate respiration, nitrogen respiration, and nitrate fixation, along with some bacterial functions with low abundances all declined to varying degrees at 150 d. This reflected that the biochemical functions are dominated by bacterial communities and their dynamic changes in response to nutrient conversion and cycling in different development stages of artificial soils. The reduced abundances and functions of nitrifying, denitrifying, and nitrogen-fixing bacteria affect the metabolism, conversion, and cycling of nitrogen in artificial soils, and the weakened nitrogen fixation may reduce soil nitrogen and affect plant growth [50]. In addition, some bacterial functions also increased in abundance, including phototrophy, cyanobacteria, oxygenic photoautotrophy, photoautotrophy, the dark oxidation of sulfur compounds, and dark sulfide oxidation. This suggests that the abundance and functions of photoautotrophic bacteria increase gradually during the development of artificial soils, which is conducive to soil carbon sequestration and organic fertility improvement [13,51]. The increase in the abundance and functions of sulfur-oxidizing bacteria promotes acid production, converts sulfides into sulfuric acid, and reduces soil pH, improving the saline–alkaline characteristics of artificial soils and plant growth [52,53]. In addition, the changes in the abundances of bacterial functions in artificial soils may also be affected by the growth, reproduction, and metabolism of other organisms during vegetation restoration, such as different types of adaptable plants, fungi, and arthropods [54,55,56,57,58,59].

## 5. Conclusions

Changes in bacterial communities, their correlations with environmental factors, and bacterial functions in the early-stage engineering simulation of red mud/phosphogypsum-based artificial soil vegetation restoration were reported for the first time in this paper. The structure of the bacterial community was simple at the beginning, where it mainly consisted of Proteobacteria, Firmicutes, and Bacteroidota, with total abundances of 74.5% and 89.3% in the two plots, respectively. The richness, diversity, and evenness of the bacterial communities all increased significantly over time (*p* < 0.05), indicating that the compositions of bacterial communities in artificial soils experience constant development, adjustment, and optimization. There were good correlations between the bacterial communities and most environmental factors (e.g., pH, W_H2O_, OM, TN, TK, AK, TP), especially in the JZ plot, which generally reflected the significant synergistic development and interaction between soil environmental quality and bacterial communities. Meanwhile, there were complex dynamic changes in the functions of bacteria during the development of artificial soils, which were mainly reflected in the decline in the abundances of chemoheterotrophy, aerobic chemoheterotrophy, and animal parasites or symbionts but increase in the abundances of phototrophy, cyanobacteria, and dark sulfide oxidation. This indicates the highly active physiological and biochemical reaction functions of bacterial communities in the development of artificial soils. It is of great significance to continuously enhance the fertility quality and ecological attributes of artificial soils. Although the experimental period in this study was short and we did not provide a comparison with natural soils, this study provides important data supplementation and scientific evidence in order to generate a deeper understanding of the changes in bacterial communities in early-stage red mud/phosphogypsum-based artificial soil vegetation restoration and their relationship with the development of artificial soil.

## Figures and Tables

**Table 1:** Material mass ratio of the artificial soil plots at the test site [11].

Name	Red Mud (%)	Phosphogypsum (%)	Distillers Grain (%)	Rice Hull (%)	Bentonite (%)	Polyacrylamide (%)
DK	53.78	35.86	/	8.96	1.34	5.38 × 10^−2^
JZ	49.36	32.91	16.45	/	1.23	4.52 × 10^−2^

**Table 2:** Bacterial α-diversity in the artificial soil plots.

Name	Sobs	Shannon (×10^−1^)	Simpson (×10^−3^)	Ace	Chao	Pielou_e (×10^−2^)	Coverage (%)
DK-30d	1057.6 ± 78.8 c	50.2 ± 2.6 c	18.9 ± 5.9 b	1416.8 ± 81.3 c	1422.6 ± 95.4 c	75.7 ± 3.0 c	98.8 ± 0.0 b
DK-150d	2360.2 ± 217.4 a	63.6 ± 0.3 a	6.3 ± 0.8 d	3126.5 ± 507.4 a	3109.4 ± 483.2 a	84.2 ± 1.3 a	97.4 ± 0.7 c
JZ-30d	620.6 ± 11.3 d	43.6 ± 0.7 d	30.7 ± 3.2 a	812.5 ± 14.9 d	801.2 ± 35.0 d	68.4 ± 1.0 d	99.4 ± 0.0 a
JZ-150d	2052.0 ± 54.9 b	59.8 ± 0.9 b	10.9 ± 2.1 c	2766.9 ± 89.7 b	2763.4 ± 118.6 b	77.1 ± 1.0 b	97.6 ± 0.1 c

Note: Different letters represent significant differences in the artificial soil plots on different days. LSD test; *p* < 0.05.

**Table 3:** Basic physicochemical indicators and major nutrients in the artificial soil plots.

**Name**	**pH**	**W_H2O_ (%)**	**OM (%)**	**CEC (cmol/kg)**	**TN (mg/kg)**
DK-30d	9.04 ± 0.02 a	34.3 ± 1.4 ab	6.3 ± 0.3 a	9.2 ± 0.1 a	337.1 ± 77.6 c
DK-150d	8.29 ± 0.04 b	32.2 ± 0.5 b	6.0 ± 0.1 b	8.6 ± 0.7 a	215.3 ± 17.3 d
JZ-30d	8.29 ± 0.01 b	34.9 ± 1.4 a	4.8 ± 0.2 c	7.2 ± 0.2 b	960.9 ± 44.5 a
JZ-150d	7.61 ± 0.02 c	29.2 ± 1.4 c	3.4 ± 0.1 d	8.5 ± 0.5 a	631.9 ± 66.7 b
**Name**	**AN (mg/kg)**	**TP (mg/kg)**	**AP (mg/kg)**	**TK (g/kg)**	**AK (mg/kg)**
DK-30d	100.9 ± 14.3 b	2273.1 ± 820.0 a	259.5 ± 3.7 a	65.0 ± 17.5 a	12.0 ± 0.3 a
DK-150d	100.9 ± 14.4 b	1873.9 ± 21.6 ab	237.7 ± 15.0 a	35.1 ± 2.9 b	10.2 ± 0.4 ab
JZ-30d	153.7 ± 8.2 a	2510.5 ± 168.8 a	232.6 ± 20.9 a	72.3 ± 7.7 a	11.1 ± 1.9 a
JZ-150d	120.0 ± 8.5 b	1379.2 ± 43.8 b	194.3 ± 17.4 b	34.8 ± 6.1 b	8.5 ± 0.2 b

Note: The data were sourced from [11]. Different letters represent significant differences in the artificial soil plots on different days. LSD test; *p* < 0.05.

## Data Availability

The original contributions presented in this study are included in the article/Supplementary Materials. Further inquiries can be directed to the corresponding authors.

## Supplementary Materials

The following supporting information can be downloaded at: https://www.mdpi.com/article/10.3390/biology14081020/s1, Table S1. The statistics of sequencing data of bacterial communities in artificial soil plots.
