# Supplementary material for: Bacterial Community Changes in Early-Stage Engineering Simulation of Red Mud/Phosphogypsum-Based Artificial Soil Vegetation Restoration
Source: Biology (Basel). 2025 Aug 8;14(8):1020. doi: 10.3390/biology14081020 (PMC12383687; doi:10.3390/biology14081020)
Supplement: Supplementary file 1 [file biology-14-01020-s001.zip › biology-3728538-supplementary.pdf]

Table S1. The statistics of sequencing data of bacterial communities in artificial soil plots.

Name	Seq_num	Base_num	Mean_length	Min_length	Max_length
DK_1(30d)	38870	16315990	419.7579	358	439
DK_2(30d)	51796	21731997	419.569	251	509
DK_3(30d)	54379	22779528	418.903	277	447
DK_4(30d)	37366	15664989	419.2311	386	479
DK_5(30d)	53738	22506455	418.8183	228	444
DK_1(150d)	52407	21870714	417.3243	233	450
DK_2(150d)	53168	22198853	417.5228	211	511
DK_3(150d)	73817	30813254	417.4276	246	484
DK_4(150d)	101925	42508171	417.0534	232	493
DK_5(150d)	37210	15545856	417.7871	398	517
JZ_1(30d)	48493	20407475	420.8334	277	442
JZ_2(30d)	49838	20956136	420.4851	266	444
JZ_3(30d)	47924	20162294	420.7139	315	506
JZ_4(30d)	40242	16944314	421.0604	262	448
JZ_5(30d)	49318	20735914	420.4533	277	431
JZ_1(150d)	53000	22138679	417.7109	251	516
JZ_2(150d)	54809	22912820	418.0485	235	515
JZ_3(150d)	55505	23173384	417.5008	246	485
JZ_4(150d)	57606	24040806	417.3316	245	460
JZ_5(150d)	247911	1.04E+08	418.7147	223	478

Note: Seq_num (sequence number), Base_num (base number), Mean_length (mean sequence length, Min_length (minimum sequence length), Max_length (maximum sequence length)
